# Does MARPE therapy have effects on intracranial pressure? a clinical study

**DOI:** 10.1186/s12903-022-02482-x

**Published:** 2022-10-19

**Authors:** Baris Baser, Merve Bolukbasi, Dilek Uzlu, Ahmet Duhan Ozbay

**Affiliations:** 1 Department of Orthodontics, Faculty of Dentistry, Karadeniz Technical University, Trabzon, Turkey; 2 Department of Ophtalmology, Faculty of Medicine, Karadeniz Technical University, Trabzon, Turkey; 3 Department of Ophtalmology, Erzurum Regional Education and Research Hospital, Erzurum, Turkey

**Keywords:** Microimplant-assisted rapid palatal expansion (MARPE), Intracranial pressure, Optic nerve sheath diameter, Ultrasonography

## Abstract

**Background:**

We aimed to evaluate possible intracranial pressure (ICP) changes caused by screw activations during active microimplant-assisted rapid palatal expansion (MARPE) therapy of post-pubertal individuals by measuring the optic nerve sheath diameter (ONSD) under ultrasonography (US) guidance.

**Methods:**

This study’s participants comprised 15 patients (7 males, 8 females) with posterior crossbite and a mean age of 16.7 years (14.25–20.08 years). The Maxillary Skeletal Expander (MSE) appliance was used to perform MARPE in all patients. Their vital signs (heart rate, mean arterial pressure (MAP), and peripheral oxygen saturation (SpO_2_)) were recorded. The ONSD was measured by US immediately before the first screw activation (T0), and the measurements were repeated 1 min (T1) and 10 min (T2) after the first activation. In the last session of active MARPE therapy, the same measurement protocol was performed as in the first activation session (T3, T4, and T5). The patients’ perceptions of pain during the screw activation were also noted at T1 and T4 using a four-category verbal rating scale (VRS-4). The significant differences among different time intervals performed with the Friedman test (for all tested variables; SpO2, MAP, Heart Rate, VRS-4 and ONSD). Spearman correlation test was used for VRS-4 and ONSD comparisons. The statistical significance level was accepted as *p* < 0.05.

**Results:**

The ONSD values ​​(T1 and T4) relatively increased within 1 min after screw activation but did not reach a statistically significant level (*p* > 0.05). There was also no significant difference between the initial (T0) and the final (T5) ONSD values ​​during the active MARPE therapy (*p* > 0.05).

**Conclusion:**

There is no changes or alterations in intracranial pressure in late adolescents during active MARPE therapy.

**Supplementary information:**

The online version contains supplementary material available at 10.1186/s12903-022-02482-x.

## Background

The positive effects of rapid expansion therapy have been proven in solving dental and other health problems, such as unilateral or bilateral posterior crossbite characterized by maxillary transverse insufficiency [1], tooth-arch size discrepancies [2], functional shift of the mandible [1], narrow smile arch [3], and sleep apnea [4]. While conventional rapid palatal expansion appliances are sufficient to achieve orthopedic expansion in the pre-pubertal period [5], this treatment is performed with microimplant-assisted rapid palatal expansion (MARPE) [6, 7] or surgically assisted rapid palatal expansion [8] in the post-pubertal period due to the interdigitating of sutures.

Maxillary expansion with MARPE method is a protocol where a strong force is applied to skeletal structures in the transverse direction by supporting only bones or both bones and teeth [9, 10]. Previous studies have shown that the accumulated force as a result of continued activations of the expansion screw increases the stress on adjacent structures, especially in the superior orbital fissure, oval foramen, round foramen, spinous foramen, and optic foramen [9, 10]. Anatomical stress centered on these structures and the displacement of bones [11] can cause injury to or change the diameter of the vessels that play an important role in supplying blood to the brain [9, 12], resulting in the stenosis of venous or cerebrospinal fluid (CSF) drainage [13] and consequently altering intracranial pressure (ICP) [14].

Intracranial pressure (ICP) is determined by the total volume of the brain, amounting from cerebrospinal fluid (CSF) and blood in the cranium [15]. According to the Monroe-Kellie doctrine [15], these components are typically well-balanced which creates a constant ICP. Thus, impairing the relations between these factors results in increased intracranial pressure [16]. Furthermore, the optic nerve, covered with a dural sheath, is a part of the central nervous system, and pressure in the infraorbital subarachnoid space surrounding it is quite similar to and is correlated with ICP [17–19]. However, recent studies have also described a non-invasive and reliable method to indicate ICP measuring optic nerve sheath diameter (ONSD) using ultrasonography (US) [17].

In the present study, we aimed to evaluate possible ICP changes undergoing MARPE treatment in post-pubertal individuals by measuring ONSD under US guidance. We also aimed to assess some parameters (SpO2, MAP, Heart Rate and pain) which related to ICP [20–22]. The hypothesis of this study is that the screw activations induce increase of ICP during active MARPE therapy.

## Methods

Approval for this study was obtained from the ethics committee of Karadeniz Technical University Faculty of Medicine Scientific the Clinic Research (24237859-263, 17/03/2021) and informed consent was obtained from all subjects and/or their legal guardian(s) for participation and publication.

The sample size was confirmed based on the previous data [17]. After considering the alpha level as 0.05, beta = 0.20, effect size = 0.9, the final total number of the required sample was found as 13. This study’s participants comprised 15 patients (7 males, 8 females), with a mean age of 16.7 years (14.25–20.08 years).

Each patient had maxillary constriction and bilateral posterior crossbite with an indication for MARPE treatment. The transversal occlusal discrepancy between upper and lower posterior teeth being ranged from 4 to 8 mm [23] and skeletal deficiency was calculated as the difference between maxillary and mandibular width [24]. This difference assisted in the diagnosis of maxillary skeletal stenosis, determination of the amount of expansion required and to finalize the expansion process. Their skeletal maturity stages were at CS4 or higher according to the Cervical Vertebral Maturation (CVM) Index.

Patients who had previous orthodontic treatment and a craniofacial syndrome were excluded from the study. The exclusion criteria were also the patients with eye diseases (diabetes-related eye conditions, retinal detachment, ocular trauma, glaucoma, or previous optic nerve atrophy), a history of previous eye surgery or neurological pathology.

**Fig. 1:**
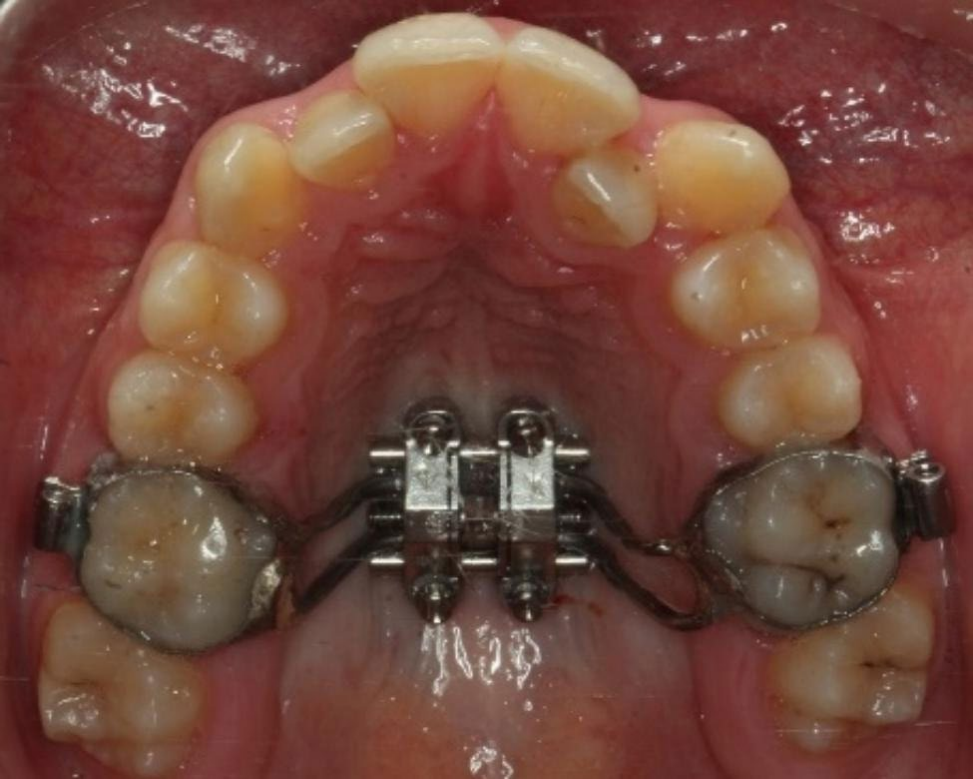
Maxillary Skeletal Expander (MSE) appliance (BioMaterials Korea, Inc.)

Maxillary Skeletal Expander (MSE) appliance (BioMaterials Korea, Inc.), introduced by Dr. Won Moon [24], was accomplished with four micro-implants with a length of 11 mm and a diameter of 1.8 mm before the bonding of orthodontic brackets. Depending on the palate width, 8-mm or 10-mm MSE’s were used and the body of the appliance was placed as posteriorly as possible, close to the junction of the hard and the soft palate, to allow the parallel opening of the suture (Fig. 1).

The expansion rate was adjusted to two rounds per day before the appearance of midline diastema and then once a day (0.20 mm per round) as described Dr. Won Moon [25]. Midline diastema between the maxillary incisors was observed in each patient.

The screws were activated by the researcher on ICP measurement days, and further activations were made by the patients or parents daily during the expansion process. When the palatal cusps of the maxillary posterior teeth were occluding with the buccal cusps of the mandibular posterior teeth, the screw was fixed in place using a flowable composite (3 M Unitek Orthodontic Products, CA, USA). The duration of active MARPE therapy was ranged from 20 to 24 days.

### Measurement of optic nerve sheath diameter

**Fig. 2:**
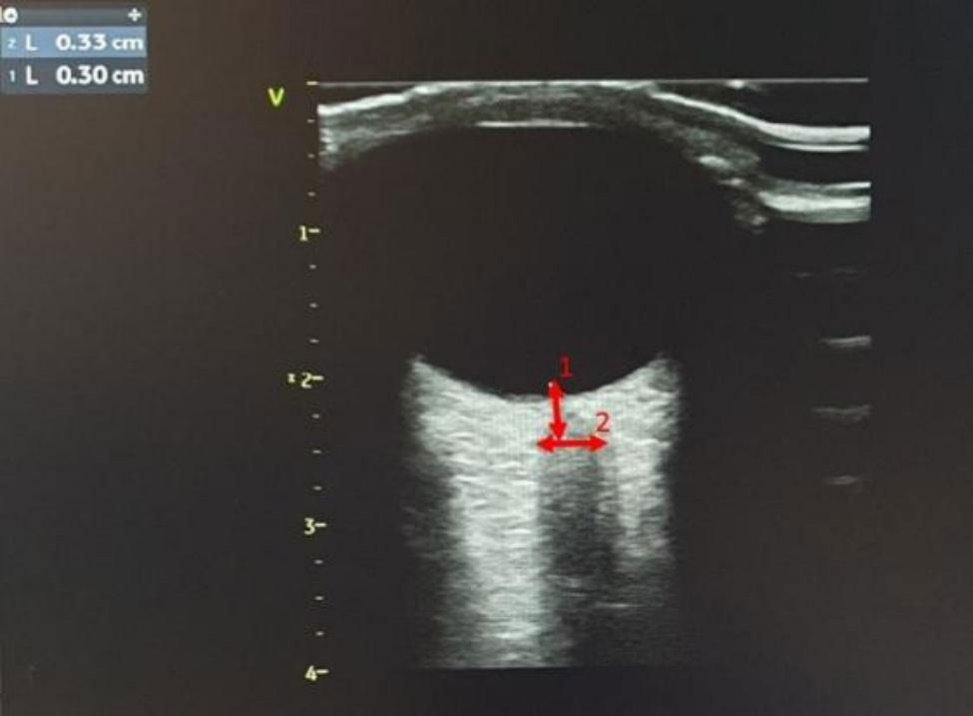
Ultrasonographic image of optic nerve sheath diameter measurement. (1. Distance behind the optic disc where the optic nerve sheath diameter (ONSD) is measured in its width, 2. ONSD measurement)

The ONSD measurements were performed in B Mode using an Aviso model US device (Quantel Medical, France) and a linear probe at a frequency of 10 MHz. Two researchers experienced in using US did all measurements by applying thick conductive ultrasound gel to the eyeballs with the patients’ closed eyelids and the probe was gently placed. The ONSD was measured at 3 mm posterior to the optic disc in the transverse axis and the vertical axis for each optic nerve (Fig. 2). The measurements were undertaken from both eyes and by two experts.

**Fig. 3:**
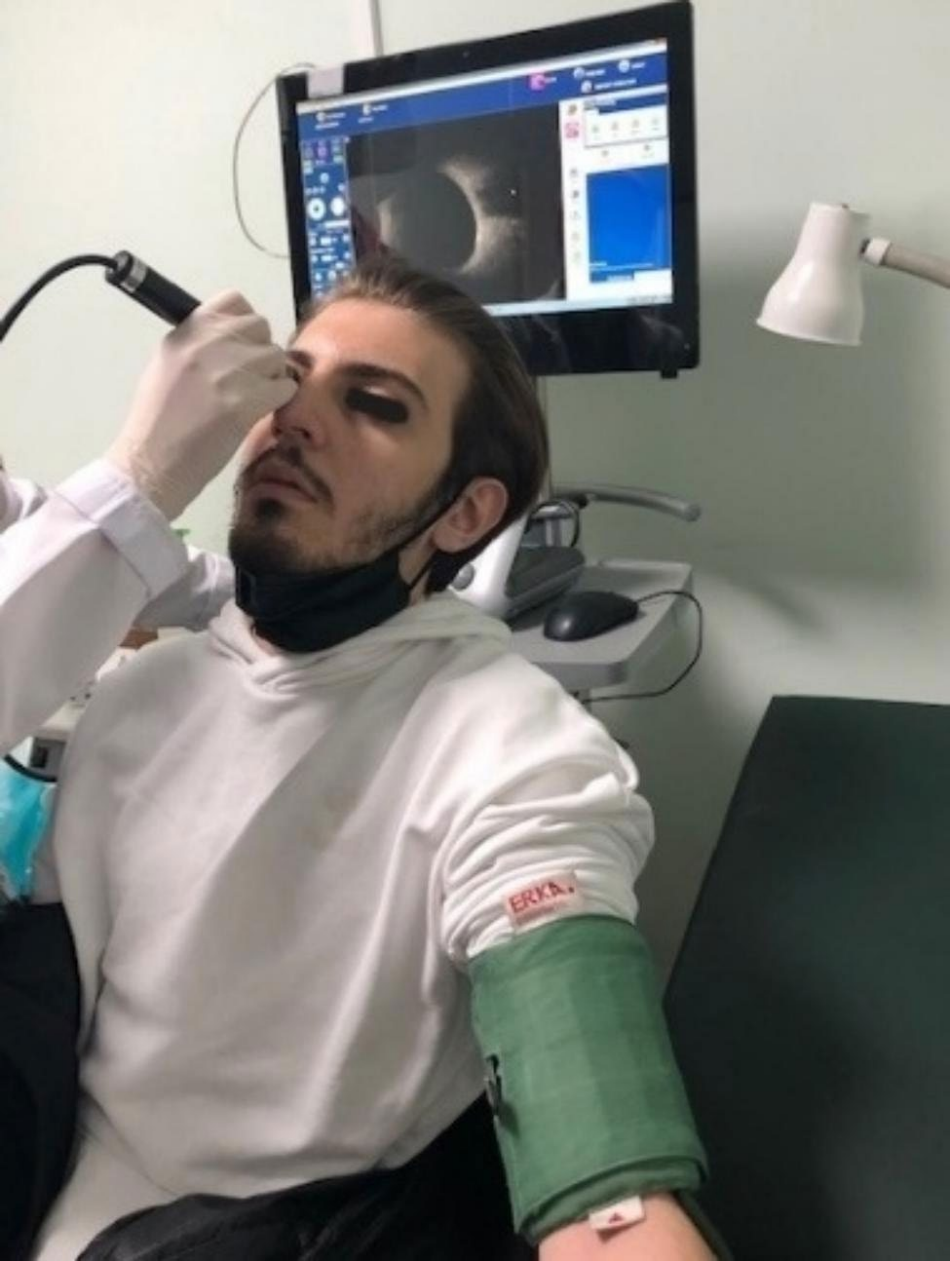
Measurement of optic nerve sheath diameter ultrasonographically

At the time of US imaging, patients were examined in the sitting position with the head being elevated to approximately 45° (Fig. 3). ONSD measurement was performed just before the first screw activation (T0). Following the first activation, measurements were repeated after 1 min (T1) and 10 min (T2). On the day active expansion completed, the same measurement procedure was performed as in the first activation appointment (T3, T4, and T5).

Hemodynamic data (heart rate, mean arterial pressure [MAP], and peripheral oxygen saturation [SpO2]) were measured at all evaluation times (T0, T1, T2, T3, T4, and T5) and recorded.

Patients’ perception of pain at screw activation times (T1 and T4) was also noted by a four-category verbal rating scale (VRS-4). Each patient was asked to report pain on a VRS-4 with self-explanatory categories (scores ranged from 0 to 4). The operators were blinded to each other’s results and the data was recorded by an independent observer.

### Statistical analysis

The Number Cruncher Statistical System (NCSS) 2007 (Kaysville, Utah, USA) was used for statistical analyses. Descriptive statistical methods (median, minimum, and maximum) were used to analyze the study’s data. The normality of data was tested with the Shapiro-Wilk test. The significant differences among different time intervals performed with the Friedman test (for all tested variables; SpO2, MAP, Heart Rate, VRS-4 and ONSD). Spearman correlation test was used for VRS-4 and ONSD comparisons. The statistical significance level was accepted as *p* < 0.05.

## Results

In total, 15 patients were included in the study. Their mean age was 16.77 ± 1.90 years. The patients’ demographic data are listed in Table 1.

**Table 1:** Demographic data of the patients

	Min – Max	Mean ± SD
Age	14 – 20	16.77 ± 1.90
Gender	Number	%
Female	8	53.3
Male	7	46.7

SD = standard deviation

The mean ONSD measurements were 5.87 ± 0.64 mm at T0, 6.05 ± 0.85 mm at T1, 6.01 ± 0.66 mm at T2, 5.83 ± 0.62 mm at T3, 5.98 ± 0.63 mm at T4, and 5.93 ± 0.58 mm at T5.

**Fig. 4:**
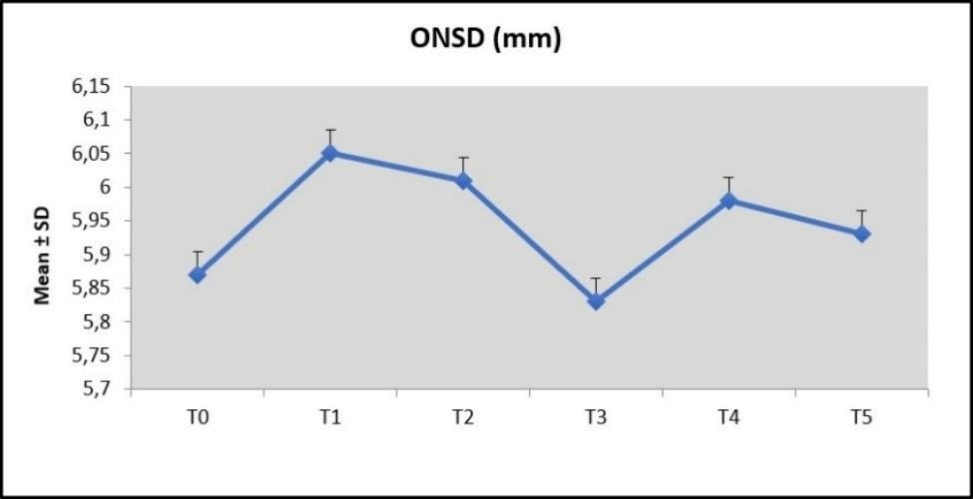
Changes in optic nerve sheath diameter (ONSD) values at each time point

The ONSD values 1 min after screw activations (T1 and T4) were relatively higher than before activations (T0 and T3). However, the relative increase between T0 and T1 and between T3 and T4 was statistically non-significant. Conversely, there was a relative decrease in the ONSD values 10 min after screw activations (T2 and T5) compared with the values 1 min after activations (T1 and T4) but the differences were also statistically non-significant (*p* > 0.05). When the ONSD values 1 and 10 min after screw activations were compared (T1–T2 and T4–T5), the results were statistically non-significant (*p* > 0.05). Furthermore, there was no significant change between initial (T0) and final (T5) ONSD values. (*p* > 0.05). The changes and comparisons of the median ONSD values at each time points ​​and the descriptive statistics are presented in Table 2; Fig. 4.

**Table 2:** Summary of optic nerve sheath diameter (ONSD) values at each time point

Time	Median (Min-Max) mm	95%CI		*p	
**T0**	5.8 (5-7.2)	5.51-6.22			
**T1**	5.8 (5-8.5)	5.58-6.51			
**T2**	5.9 (5-7.8)	5.64-6.37		>0.05	
**T3**	5.6 (4.7-7)	5.48-6.16			
**T4**	5.7 (5-6.9)	5.63-6.32			
**T5**	5.8 (5-7)	5.60-6.24			

*Friedman test

When the heart rate, SpO_2_, and MAP values of the patients were compared with the values of ONSD at T0, T1, T2, T3, T4 and T5, the differences were statistically insignificant (*p* > 0.05). A summary of the potential effects of hemodynamic parameters on ICP is depicted in Table 3.

**Table 3:** Hemodynamic parameters associated with intracranial pressure at each time point

Measurement	T0	T1	T2	T3	T4	T5	*P
**median (min-max)**							
**SpO2 (%)**	98 (97-99)	98 (97-99)	98 (90-99)	98 (91-99)	98 (96-99)	98 (96-99)	
**Heart Rate**	93 (69-142)	94 (59-131)	90 (65-128)	90 (60-130)	89 (71-120)	88 (68-121	>0.05
**MAP (mmHg)**	93.3(73-103)	94(59-131)	90(65-128)	81.6(70-124.3)	76.6(63-103.3)	83.3(60-96.3)	

*Friedman test (for all parameters)

The correlation was also insignificant between the changes in pain scores and ONSD values both at initial (T0–T1) and the final (T3–T4) screw activations of active MARPE therapy (*p* > 0.05). The correlation between pain and ONSD changes are presented in Table 4.

**Table 4:** Evaluation of ONSD changes according to pain intensity changes

	Pain		Pain	
	**T0-T1**		**T3-T4**	
**ONSD**	**r**	**p**	**r**	**p**
**T0-T1**	0.359	0.189	0.296	0.285
**T3-T4**	0.273	0.325	0.182	0.517

r: Spearman Correlation Coefficient, T0-T1: initial activation of the screw, T3-T4: final activation of the screw, no statistically significant correlation was found between the changes in pain intensity of the cases and the changes in ONSD (p > 0.05)

## Discussion

In the present study, changes in ICP during screw activations were prospectively evaluated with US-guided ONSD measurements in late adolescent patients undergoing MARPE therapy. Although the current literature suggests that palatal expansion devices may have extensive craniofacial outcomes, this information is often overlooked in clinical practice [26]. In two studies, it was described that serious complications could be in the cranial base after rapid expansion [27, 28]. However, Sun et al. proved that cumulative expansion forces applied to the maxilla also induced a tension beyond the physiological limit in peripheral maxillary sutures [26]. Although MARPE has shown evidence of clinical success [7, 29, 30]. It is difficult to predict exactly what occurs physiologically [31]. Additionally, it is still unclear that transmitted expansion forces how to affect brain hemodynamics and it has not yet been extensively investigated [32]. Thus, we aimed to evaluate possible ICP changes caused by MARPE therapy in post-pubertal individuals by measuring ONSD under US guidance.

Some studies have emphasized the importance of age in palatal expansion procedures [33]. Resistance to skeletal separation increases after the pubertal growth spurt; therefore, significant suture separation cannot be expected with tooth-supported expanders [34]. Previous studies have revealed a significant relation between the Cervical Vertebral Maturation (CVM) index and suture maturation [35].

Although a review of the literature demonstrates the extensive effects of the forces produced during the rapid expansion of the palate, little is known about the exact nature of these forces and how they are transmitted through the craniofacial complex [12, 36–38]. During rapid expansion, the pterygoid processes are strongly bent laterally, resulting in yet-to-be-clarified effects on the sphenoid and foramina, through which important nerve and vascular structures pass, as well as on the entire cranial base [39]. While neural structures are not expected to be damaged due to relatively low stress in juvenile cranial structures during rapid maxillary expansion, this damage cannot be ignored due to much greater stresses caused by increased stiffness and decreased elasticity in skeletal structures with age [9, 40]. In a case report, researchers recommended that clinicians should consider pseudotumor cerebri syndrome (PTCS), as the syndrome may result in headache and/or visual disturbances during the expansion process [14]. Similarly, Lanigan and Mintz reported that partial oculomotor nerve paralysis occurred in an adult after surgically assisted rapid maxillary expansion without separating the pterygomaxillary junction [41]. A study conducted in monkeys showed that changes occurred in the surrounding structures as a result of the rapid expansion of the midpalatal suture [38]. Although prior studies have primarily focused on effects of forces on craniofacial structures in patients of expansion treatment, the possible secondary results on the intracranial vascular compartment, brain hemodynamics, and intracranial pressure are yet undefined [9, 42].

Intracranial pressure (ICP) is determined by the total volume of the brain, amounting from cerebrospinal fluid (CSF) and blood in the cranium [15]. According to the Monroe-Kellie doctrine [15], these components are typically well-balanced which creates a constant ICP. Thus, impairing the relations between these factors results in increased intracranial pressure [16]. Furthermore, both optic nerves are surrounded by the dural sheath, which is an extension of the meninges protruding into the orbit [43]. This causes similar cerebrospinal fluid pressure changes between the intracranial and the infraorbital subarachnoid spaces, suggesting that sheath dilatation reflects increased ICP [43, 44]. Thus, possible ICP changes were evaluated by measuring ONSD under US guidance.

Various methods such as Computed Tomography (CT), Magnetic Resonance Imaging (MRI), ICP monitoring, and lumbar puncture are used for the measurement of ICP [45]. However, these methods have limitations in terms of invasiveness, contraindications, radiation exposure, availability, and requirement for patient transport [43, 46]. In recent years, the measurement of ONSD using trans orbital US has been described as a non-invasive alternative method for assessing ICP in children and adults, with no complications observed during post-operative follow-up [17]. A recent systematic review and meta-analysis of studies comparing ONSD values on US with direct ICP monitoring showed a strong correlation between the two [47]. In the present study, ONSD was measured by US, a rapid and non-invasive method, to evaluate the effect of MARPE on ICP, and no complications were observed in post-operative follow-up.

Although studies have shown a good correlation between the measurements from the left and the right eyes [48], in the present study, the mean value of ONSD measurements were used from both eyes to exclude individual anatomical variations and the possibility of any unilateral pathology. It is also very important for the results’ reliability that the diagnostic methods used are reproducible and independent of the operator performing the measurements [48]. Regarding the inter-observer reliability, Lochner et al. found a strong correlation between the measurements of two operators [49]. Similarly, Ballantyne et al. also found that sonographic measurement of ONSD is a reproducible technique with low intra- and inter-observer variation [50]. Therefore, the measurements were undertaken by two experts to minimize operator-related differences.

In the present study, the ONSD values 1 min after screw activations (T1 and T4) were relatively higher than before activations (T0 and T3). However, the relative increase between T0 and T1 and between T3 and T4 was statistically non-significant. Although the pathophysiology of the increase in ICP is not clearly known, we consider that the relative increase may have occurred as a result of varying degrees of stress, minimal bone displacement, and cerebral hemodynamic changes in the intracranial region. However, some studies suggest that major changes in ICP are required to cause significant changes in ONSD [51]. Conversely, there was a relative decrease in the ONSD values ​​10 min after screw activations (T2 and T5) compared with the values 1 min after activations (T1 and T4) but the differences ​​were also statistically non-significant (*p* > 0.05). We suspect that it is likely due to the autoregulatory capacity and compensation mechanism of the brain [52]. Correspondingly, a previous study showed that the Boyle-Davis mouth plug increased the ONSD on US during adenotonsillectomy operation and back to the normal after the plug was removed, possibly due to autoregulation [17]. In another study imaging CT, cerebral blood flow was shown to increase at the early stages of rapid palatal expansion therapy and then return to normal [12]. Additionally, the relationship between initial (T0) and final (T5) ONSD values was found to be insignificantly correlated (*p* > 0.05). Our study shows that the optic nerve sheath diameter, which indicates ICP, relatively increases with the screw activation and back to its basal value at the end of the active expansion therapy. Furthermore, the repetitive activations do not affect this process. We suspect that relatively raising in pressure balances via components of ICP. Even though relatively increases with the screw activation, our hypothesis was rejected according to our findings.

Changes in blood pressure, pulse, and respiration are clinically important since they cause ICP alteration [20, 21]. Raised ICP can significantly reduce CBF, leading to ischemia and cell death. [21]. In the early phase of cerebral ischemia, vasomotor centers are stimulated and systolic pressure increases to maintain CBF. [20]. This is accompanied by a slow heartbeat and an irregular respiratory rhythm [53]. Low blood oxygen saturation (hypoxia) and high blood carbon dioxide levels (hypercarbia) cause to increase blood volume resulting undesirable elevation of ICP due to the dilation of vessels connected to the brain [53]. To account for all of these components which relate to ICP, changes in heart rate (HR), oxygen saturation (SpO2), and mean arterial pressure (MAP) values were evaluated independently, and their relation with ICP was not found to be statistically significant (p > 0.05). These findings confirmed that all hemodynamic parameters were stable at each time points and similar with the results of ONSD measurement.

Studies have shown that pain elevates ICP and increases blood pressure, resulting in respiratory irregularity [22, 54]. In our study, the patients’ perceptions of pain were also evaluated with the VRS-4 at screw activation times (T1 and T4). Pain scores (T0–T1 and T3–T4) were non-significant at both the initial and the final appointments of active expansion treatment (*p* > 0.05). Therefore, we conclude that the pain had no effects in ICP as hemodynamic parameters at the activation times.

As a result of all these findings, we can state that active MARPE therapy has no significant effect on ICP. However; long-term ICP changes cannot be predicted based on our data. When tension-related bone changes are considered, the duration of therapy is an important variable for mechanical stress [55]; therefore, longer-term studies are needed. There is also a need for more comprehensive studies with a larger sample size to ensure that physicians apply MARPE therapy without any concerns about ICP, especially in patients with intracranial pathologies, which has not yet been evaluated. In addition to the limitations of the study, optic nerve sheath is not always strictly circular and its extensibility may show varies each for individuals while US imaging. Moreover, lack of control group and US is to be an operator dependent imaging method are also limitations.

## Conclusions

There is no changes or alterations in intracranial pressure in late adolescents undergoing MARPE therapy due to possible autoregulation of the brain but it may have some risks in patients with intracranial pathology. Thus, we suggest that clinicians should consider the risks of raised ICP and to research in further studies.

## Electronic supplementary material

Below is the link to the electronic supplementary material.

Supplementary Material 1

## Data Availability

All data generated or analysed during this study are included in supplementary information files.

## Declarations

### Author information

1 Department of Orthodontics, Faculty of Dentistry, Karadeniz Technical University, Trabzon, Turkey. 2 Department of Ophtalmology, Faculty of Medicine, Karadeniz Technical University, Trabzon, Turkey. 3 Department of Ophtalmology, Erzurum Regional Education and Research Hospital,Turkey.

## Abbreviations

RME: Rapid Maxillary Expansion
ONSD: Optic Nerve Sheath Diameter
US: Ultrasound
CSF: Cerebrospinal Fluid
CBF: Cerebral Blood Flow
CBV: Cerebral Blood Volume
MRI: Magnetic Resonance İmaging
PTCS: Pseudotumor Cerebri Syndrome
ICP: Intracranial Pressure
CBT: Computerized Brain Tomography
CVM: Cervical Vertebral Maturation
MAP: Mean Arterial Pressure
SpO2: Peripheral Oxygen Saturation
VRS: Verbal Rating Scale
